# “Art and Psyche Festival”: Utilizing the power of art against the stigma around mental illness

**DOI:** 10.3389/fpsyt.2022.1027316

**Published:** 2023-01-26

**Authors:** Arman Moeenrad, Farah Alizadeh Jouimandi, Nasim Kamalahmadi, Aida Ghofrani Ivari, Samin Davoody, Hossein Mohaddes Ardabili, Mojtaba Ghalandarzadeh, Farideh Sinichi, Bahareh Hakimi, Zahra Rajaei, Narjes Sahebzadeh, Anahita Arabi, Sahar Omidvar Tehrani, Zohreh Mahdianpour, Maedeh Kamrani, Fateme Farhoudi, Ali Saghebi, Mohammadreza Fayazi Bordbar, Ali Talaei

**Affiliations:** ^1^Psychiatry and Behavioral Sciences Research Center, Mashhad University of Medical Science, Mashhad, Iran; ^2^Student Research Committee, Faculty of Medicine, Mashhad University of Medical Sciences, Mashhad, Iran; ^3^Student Research Committee, School of Medicine, Shahid Beheshti University of Medical Sciences, Tehran, Iran; ^4^Research Center for Psychiatry and Behavior Science, Shiraz University of Medical Sciences, Shiraz, Fars, Iran

**Keywords:** art, festival, stigma, mental health, Iran, public engagement, public health

## 1. Introduction

Despite the great scientific advances in psychiatry and its extensive reflection on society, stigma toward mental health conditions, patients, and even hospitals remains dominant (1). Mental health issues are a double-edged sword; The symptoms, distress, and disability that interfere with individuals' daily lives, along with the surrounding stigma, can make a challenging situation. Stigma toward mental health conditions can be defined as cognitive and behavioral constructs of stereotypes, prejudice, and discrimination (2). People with mental health issues experience different types of stigma from various sources, including public stigma, self-stigma, and structural stigma (2).

Stigmatization attitudes toward psychiatric disorders prevent patients from seeking professional psychological help (3). The fact that individuals with mental health disorders such as depression (4), anxiety (5), and schizophrenia (6) are afraid of being socially labeled by stigmatizing words such as insane or crazy continues to place barriers against obtaining help (7). The results of the Mental Health Million project, which is a survey on global mental wellbeing launched by Sapien Labs, revealed that in 2021, more than 50% of people with clinical mental health risks did not seek psychological help. It also stated that 25% of those not seeking help cited stigma as the leading cause (8).

The stigma toward mental health disorders and help-seeking behavior has altered over time. The study conducted by Leach et al. in 2009 showed a decline in the stigma of seeking mental health counseling and a rise in the acceptance of and the need for mental health services over the past 20 years in Egypt, a Muslim community with a traditional context (9). Another US population-based study published in 2021 reported a significant decrease in public stigma regarding depression but not schizophrenia (10). Taking the progress in destigmatization into account, it seems that the stigma around mental health issues has not been eradicated; rather, some less severe mental health conditions and treatments have become less stigmatized as a result of the destigmatizing efforts (7).

Fighting against stigma is a challenging road. To overcome this challenge, efforts must be made not only on personal levels but also on public levels (2). Various approaches to changing the stigma of mental health issues could be grouped into three categories: *protest, education*, and *contact*. **Protest** aims to suppress stigmatizing attitudes by highlighting stigma's injustices and confronting the offenders for negative attitudes and behaviors (2, 11). **Education** aims to raise public awareness and tends to have its best effect among teens and adolescents (2, 12). As another method of fighting against stigma, the literature suggests that interpersonal **contact** with someone with a mental health issue reduces stigma more than either protest or education approaches (2, 11, 12). Anti-stigma movements try to implement these strategies by designing novel, creative, and socially attractive plans and mediums. Among these, art has shown a promising capacity for public engagement in social anti-stigma campaigns.

In this article, we highlight the importance of stigma related to mental health issues as a global problem and review some anti-stigma efforts in the literature. Then, we briefly discuss the relationship between art and mental health and present our experience in an anti-stigma model attracting social attention to psychiatric issues using art as the primary medium.

## 2. The association between psyche and art

Art can be defined as any means for the expression of individual and social values through concrete and artistic activities and processes (13). The rehabilitating impact of art on both mental health and life satisfaction is well-studied (14–17). This can be in the form of music (16, 18), visual arts (19), dance and movement programs (20), expressive writing such as journaling (21), and other alternatives.

Art can also be addressed as a tool for increasing public awareness about mental health and reducing the stigma toward mental health issues (22). Different forms of art, such as visual, literary, and performing arts, can be used as educational approaches to improve relatability, interactivity, and engagement (13). Using multiple art forms, especially in the form of carefully programmed, collaborative, and community-based festivals, can reduce discriminatory behavior toward people living with mental health problems and positively impact stigma around mental issues by constructing shared meanings and engaging audiences on an emotional level (23). Table 1 briefly reviews a number of art-related festivals around the world aiming to increase awareness about mental health disorders and fight the surrounding stigma. Mentioned art-related events were selected using the broad search in Pubmed, Scopus, and Embase. The search strategy was the combination of keywords: (festival OR campaign) AND art AND mental AND stigma. The search yielded a total of 22 results, excluding the duplicates. Finally, seven studies were found to be eligible for this brief review table (24–30).

**Table 1:** A brief review of a number of destigmatizing art festivals with the main goal of increasing awareness about mental health disorders^*^.

**Campaign/ festival**	**Aim**	**Target population**	**Art tools utilized**	**Place**	**Year conducted**	**References**
Mental health arts and film festival	To end mental health stigma and discrimination	Citizens of Glasgow and Lanarkshire	Film, theater, comedy, concert, community event, debate, discussion, and workshop	Scotland	2007	(24)
“Wellness and Talking Wellness”	To communicate effectively and to decrease the stigma of depression	African-Americans in Los Angles, USA	Poetry, film, and photography	African-American region of the USA	2004–2005	(25)
“AUSNAHME|ZUSTAND” (State of Emergency)	To decrease the stigma and social distance of the audience toward people with mental illness	Adolescents	Film, documentary	Germany	2008–2010	(26)
“Open the Doors”	To improve public knowledge and to reduce the stigma toward schizophrenia and schizophreniform disorders	General and specific target groups such as students, teachers, health professionals, police, and journalists	Workshop, theater, painting, film	27 countries	1999-present	(27)
Trapped in the Labyrinth	To challenge stigma and increase awareness and understanding of mental illness	General public audiences	Drama and devised performance in theater	United Kingdom	2016	(28)
“Citizenship, Compassion, the Arts” of Hong-Kong	To help with destigmatizing and to increase “understanding and support” for people living with mental illness	Individuals with mental illness and the general public	Art exhibition and art-making workshop	Hong Kong	–	(29)
“Altered States of Consciousness”	To increase public awareness of psychotic experiences	Respondents to advertisements in South-East London, local artists, visitors to the exhibition, the production team	Workshop with people with lived experience, trained actors, artwork, voice hearing simulation, video installation	UK	2017	(30)

^*^Mentioned campaigns and festivals were selected using the broad search in Pubmed, Scopus, and Embase. The search strategy was the combination of keywords: (festival OR campaign) AND art AND mental AND stigma. The search yielded a total of 22 results, excluding the duplicates. Finally, seven studies were eligible for this brief review table.

One of the well-known worldwide artistic festivals aiming to fight mental health-related stigma is the Scottish mental health art festival (SMHAF). SMHAF is an annual festival that aims to fight mental health problems and their surrounding stigma using different types of arts, from music, film, and visual art to theater, dance, and literature (31). Potash et al. stated in an article that the aforementioned art festivals could positively affect the stigma of mental health disorders (29).

In another study, Riches et al. aimed to raise the awareness of the general population and correspondingly reduce the stigma toward mental health issues through a mental health-awareness audio tour co-produced and narrated by young adults with relevant lived experience. Gallery visitors were led on ten stops through the gallery, focusing on artworks, challenging common myths about mental health, and inviting visitors to consider their personal views. The tour increased positive attitudes, indicating the feasibility of arts-based interventions in reducing stigma (32).

Another project, reported by Riches et al., aimed to raise the general population's awareness and reduce the stigma toward psychotic experiences by holding an art exhibition. The developers tried to create a semi-psychotic experience for the visitors by using voice-hearing simulations and video installations with the help of people who have lived the situation and based on their experience. The results showed that the exhibition achieved its aim by raising awareness about mental health (30).

Similarly, the BIG Anxiety Project, a citizen science art project performed in Sydney, Australia, utilized arts to inspect public attitudes toward anxiety. People represented their subjective anxiety experiences through various types of art engagement, such as installation. The project not only led to enhanced knowledge of mental health but also to spreading public participation in research that establish connections to communities (33).

All aforementioned studies confirm that the efficient use of art could help reduce the stigma and raise awareness of mental health issues.

## 3. “Art and Psyche Festival”

Studies have shown moderate to high levels of stigma toward mental health issues in Iran (34–36), a middle eastern country that lacks comprehensive plans to reduce the stigma (37). Taghva et al., in an article aimed to explore the opinions of stakeholders of mental health about the strategies to reduce the stigma toward people with mental disorders in Iran, suggested that cultural, artistic, or athletic festivals with a diverse range of general or specific audiences are of potency to reduce stigma (38). As mentioned in earlier paragraphs, various approaches to changing the stigma of mental health issues could be grouped into three categories: protest, education, and contact (2). Art festivals can offer anti-stigma means in all these categories (23, 39); therefore, considering the current situation of stigmatized attitudes locally, we decided to run an art festival focusing on mental health problems.

The idea of running the “Art and Psyche Festival” was first mentioned during the informal gatherings of the psychiatry department faculties and then developed gradually. Initially, five psychiatry faculties of the Mashhad University of Medical Science (the five latter authors) developed the idea of running the ‘Art and Psyche Festival” in the Ibn-e-Sina psychiatric hospital in Mashhad, Iran. They invited psychiatry residents to join them in organizing a committee in august 2019. Eleven psychiatry residents and three medical students formed an executive team. They started to review the available literature on art festivals and destigmatizing programs around mental health issues. They tried to expand their connections and links in many informal gatherings with well-known artists. They also managed to attract financial support from charity departments and governmental/non-governmental organizations. The organizing team began to share the invitation to the art festival on social media. As a result of these activities, many individuals and groups, including the general population, artists, art associations, public health charity organizations, private companies, related governmental organizations, NGOs, and authorities, actively got involved in participating and supporting the festival. The executive team organized and categorized the received artistic documents and forwarded them to the jury to rank and select the awardees.

The main goal of the art and psyche festival was to develop an artistic visual exhibition on the campus of a psychiatric hospital to invite every citizen to come to and experience its environment with the aim of challenging some traditional stigmatized beliefs about mental illness and psychiatric hospitals. This festival, with its competitive artistic nature, also encouraged artists to pay closer attention to mental health issues and their related stigmas. Additionally, it aimed to set the ground and promote future research on the potential risks and benefits of such measures in raising mental health and reducing related stigma among communities.

Since 2019, two online mental health art festivals have been organized with a jury committee of well-known artists, critics, and psychiatrists. The first festival, which was a national festival, was performed in three different fields of art, including photography, short movies, and short stories. The participants were asked to send their works of art in the field of mental health diseases in people's daily life. A total of 1,309 artworks (847 photographs, 342 short stories, and 120 films) were received from Iranian artists. The artworks were then reviewed and evaluated by the jury, and the ones with the highest scores in each field were awarded.

**Figure 1:**
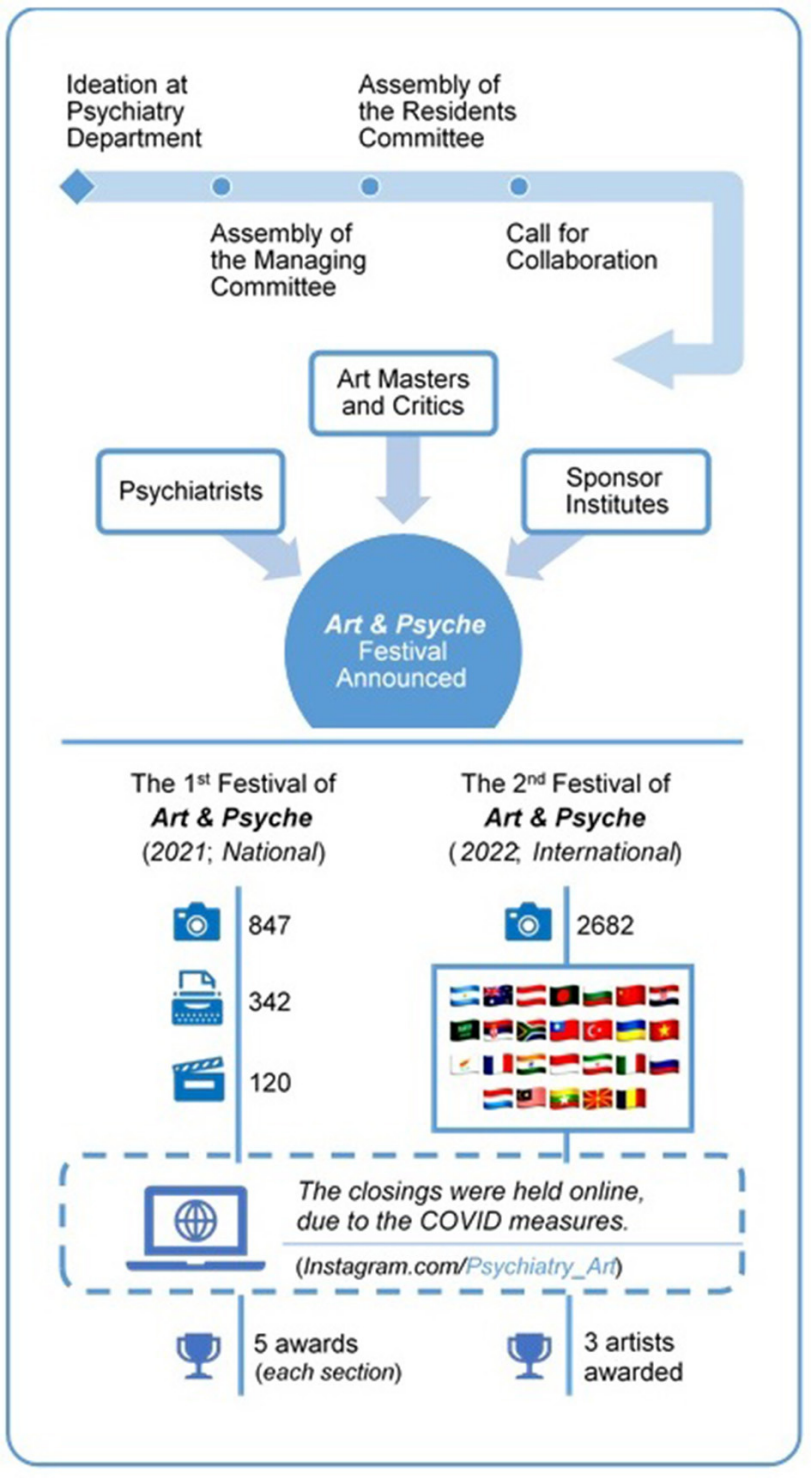
Process of organizing the “Art & Psyche Festival” from ideation until the closing event.

In the second festival, we tried to publicize the event globally on social media and invite participants from other countries. In this way, we managed to attract artists from different nationalities and promote the second festival to an international one. The second festival was focused only on photography and covered the following topics: psychological trauma during the COVID-19 pandemic, psychiatric illness and survival, marginalization and mental disorders, positive parenting, effective communication, psychological resilience, acceptance of differences, social emergencies, economy and mental health problems, children and their psychological world, child abuse, and mental health in vulnerable women. Two thousand six hundred and eighty-two photos from 26 countries on all continents were received. The three most participating countries were Iran (1,540 photos), Vietnam (281 photos), and Turkey (238 photos). Artworks receiving the highest scores from the jury were awarded a cash prize. Figure 1 visually summarizes the festivals' ideation and execution process; also, the *artwork booklets are available at: https://doi.org/10.6084/m9.figshare.21747722.v1 and https://doi.org/10.6084/m9.figshare.21747740.v1*.

This festival is among the first faculty-based festivals relating art and psychiatry in Eastern Mediterranean Region. Turning from a national event to an international one in its second year, and doubling the number of artworks, reflects its potential to become a worldwide movement against mental health issues. This festival planned to provide an anti-stigma model coordinated with active public participation, despite some prior art festivals where participants were only visitors of the artworks (30, 32).

As discussed previously, *education, protest*, and interpersonal *contact* are three major approaches that can be used to fight the stigma surrounding mental health issues (2, 11). In this festival, we tried to use art as a tool not only for raising awareness but also for connecting participants (citizens and artists) with mental health issues and individuals with such problems. We encouraged participants to depict people with mental health problems and their issues in daily routine life using their artistic and creative perspectives.

According to the available literature, we hypothesize that such an art festival can act as a multi-potent anti-stigma package providing all three main approaches to fighting against mental health stigma, including *protest* (highlighting routine challenges of people with mental illness and how stigma can even worsen their situation), *education* (raising public awareness and implicit psychoeducation about mental illness and its related challenges), and *contact* (connecting citizens with psychiatric care facilities and individuals with mental health issues).

We assume that the anti-stigmatization impacts of this art festival could be more significant if we could perform the closing ceremony at the psychiatry hospital yard, where more people get the chance to be directly engaged; however, due to COVID-19 confinements and limitations, the closing ceremony was performed online. Another limitation of this art festival was the lack of quantitative/qualitative research to support the hypothetical role of this art festival in reducing the stigma related to mental health issues. Therefore, we contemplate re-conducting it in a more intense research design evaluating its potential risks and benefits for the fight against public mental health stigma.

## 4. Conclusion

Although organizing two “*Art and Psych*” festivals in a developing country with moderate to high stigma toward mental health issues (34–36) could not achieve our ultimate goal of gathering people in an art exhibition held in a psychiatric hospital due to COVID-19 limitations, it was a successful experience in gathering social attention and support. Hypothesizing the facilitating role of art in destigmatizing psychiatric disorders, we contemplate running the third festival integrating research methods to support this opinion. We did not assess the actual anti-stigma effect of this festival; however, according to the available body of literature, we firmly believe that popularizing mental health issues using art as an attractive medium and involving social organizations will be effective in destigmatizing psychiatric disorders.

## Author contributions

AS, AT, FF, MF, and MK developed the central idea of the event, led the team through each stage, and collaborated with the jury for the art piece evaluation. AA, AM, BH, FA, FS, HM, MG, NK, NS, SO, ZM, and ZR contributed to the development of the event concept, along with participation in the festival's executive committee. AM, FS, NK, and MG contributed to the public relations and the public promotion of the event and the artworks' reception and indexing. FA contributed to the coordination with various organizations and institutes. AM and NK collaborated to prepare, design, and publish the festival books. SD, AG, HM, and NK composed the manuscript draft and collaborated with the AS, AT, FF, MF, MK, AA, AM, BH, FA, FS, MG, NS, SO, ZM, and ZR for its finalization. AM prepared the figure supplied with the manuscript. SD contributed in designing Table 1. All authors contributed to the article and approved the submitted version.
